# Sustainable Dual Cross-Linking Strategy of Lignin with Oxazoline-Modified Nitrile Rubber for Mechanically Robust Elastomeric Composites

**DOI:** 10.1021/acsomega.6c04348

**Published:** 2026-08-04

**Authors:** Kunal Manna, Samson Gnanadass, Jaipal Gupta, James J. C. Busfield, Biqiong Chen, Ton Peijs, Chaoying Wan

**Affiliations:** † Warwick Manufacturing Group (WMG), 2707 University of Warwick, Coventry CV4 7AL, U.K.; ‡ School of Engineering and Materials Science, 4617 Queen Mary University of London, Mile End Road, London E1 4NS, U.K.; ⊥ Department of Chemistry, University of Liverpool, Crown Street, Liverpool L69 7ZD, U.K.

## Abstract

Lignin, the most abundant aromatic biopolymer in nature, represents a highly promising renewable feedstock for the development of value-added polymer composites. However, its inherently poor compatibility with elastomeric matrices limits its effective utilization in high-performance and multifunctional lignin-filled systems. In this study, we report a cost-effective and sustainable cross-linking strategy to compatibilize and chemically integrate lignin within an acrylonitrile butadiene rubber (NBR) matrix through interfacial oxazoline chemistry, without the use of conventional sulfur- or peroxide-based curing agents. A small fraction of the pendant nitrile (−C≡N) groups of NBR were selectively converted into oxazoline rings via reactive melt compounding. Furthermore, interfacial compatibility was enhanced by incorporating 4 phr ZnCl_2_, which promotes strong metal–ligand coordination interactions between Zn^2+^ ions and oxazoline functionalities in the modified NBR. This approach results in the formation of a double cross-linked network comprising (i) permanent covalent linkages generated through oxazoline ring-opening reactions initiated by nucleophilic attack of lignin phenolic hydroxyl (−OH) groups and (ii) dynamic sacrificial networks formed via Zn^2+^–oxazoline, Zn^2+^–cyano, and Zn^2+^–phenolic hydroxyl/carboxylate coordination interactions involving residual nitrile groups of NBR and phenolic hydroxyl/Carboxylic acid groups of lignin. The presence of this dual cross-linking architecture significantly enhances mechanical performance. At 60 phr lignin loading, the oxazoline-modified NBR/ZnCl_2_ composite exhibits an approximately 129% increase in tensile strength relative to the NBR/ZnCl_2_ system without oxazoline modification. In contrast, the unmodified NBR/ZnCl_2_ composite containing the same lignin loading shows a comparatively lower tensile strength improvement of 86%. The pronounced reinforcing effect of lignin in the oxazoline-modified system is attributed to its dual function as both a reinforcing filler and reactive cross-linking component, as well as to the synergistic contribution of permanent covalent bonds and reversible coordination interactions within the double cross-linked network.

## Graphical abstract

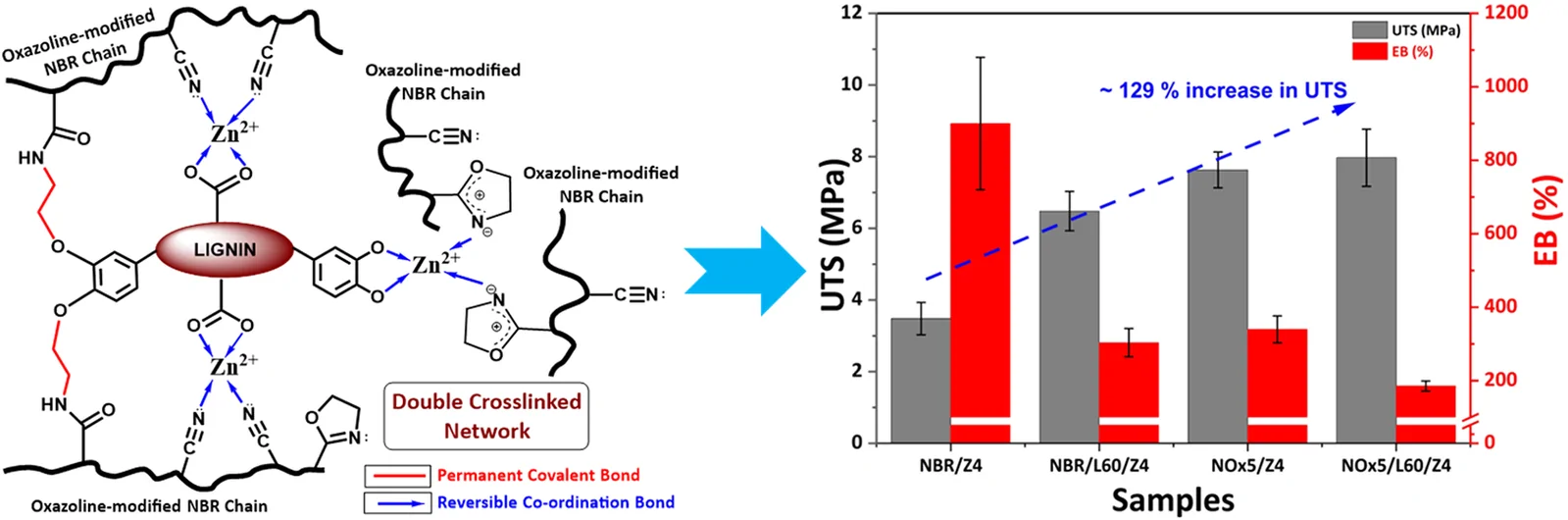

## Introduction

Recently, the utilization of elastomeric composites has gained significant popularity as advanced material systems due to their unique combination of deformability and durability for their multifaceted applications in the diverse field of automotive, biomedical, aerospace, agriculture, sports, and ocean engineering.^1^ However, the sustainable development of such advanced elastomeric composites necessitates manufacturing mechanically robust, self-healing elastomeric composites derived from biobased feedstocks to ensure durability, extend the service life, and reduce reliance on fossil resources.^1^ Therefore, to avoid concerns about the environmental impact of nonrenewable fossil resources, the building blocks of such elastomeric composites, when produced from low-cost renewable feedstocks, have been experiencing rapid and widespread adoption due to their lower production cost and nonpetrochemical origin.^2^

In this regard, lignin has been considered an important and promising option for widespread adoption and high-volume use, as it is viewed as a much more sustainable feedstock. Several sustainable lignin-based elastomeric composites have been reported utilizing lignin as a hard plastic phase.^2^ Not only that, by virtue of the cost-effectiveness, antioxidant property, and biodegradable nature of lignin, it has been explored as a reinforcing filler as a partial replacement of the traditional inorganic and carbonaceous fillers, such as silica and carbon black (CB), in elastomers.^3–5^ However, due to the polar nature of lignin and strong intra- and intermolecular hydrogen-bonding interactions, it tends to aggregate in nonpolar elastomer matrices. Such incompatibility of polar lignin with a nonpolar elastomer matrix also produces an inefficient load transfer at the polymer–filler interfaces. This hinders high-volume manufacturing of lignin-based polymer composites.^6^

Therefore, to combat the weak reinforcing nature of lignin and its poor miscibility with nonpolar elastomer matrices, considerable efforts have already been made to further chemically modify lignin, exploring different chemistries, such as silylation,^7,8^ esterification,^9,10^ and alkylation,^11,12^ to make it compatible with the host matrix. Although such additional steps can improve the compatibility of lignin with a nonpolar polymer to a certain extent, there are still many drawbacks, such as a lengthy duration of the modification reactions, involvement of an excess of organic solvents, and energy-intensive processes, making industrial application for these materials both expensive and unsustainable. Even after such surface modification of lignin, the reported improvements in filler–matrix interfacial interactions are still rather modest.^13–15^

To overcome this, nature-inspired sacrificial bonds, including reversible dynamic noncovalent bonds formed by hydrogen bonds, ionic bonds, and/or coordination bonds, have been introduced at the interface of the filler and matrix.^16,17^ When the sacrificial bonds in the form of metal–ligand coordination or ionomers are incorporated into the interphase between the elastomer and fillers, the interfacial interactions can be enhanced, promoting the filler dispersion and improved stress transfer together with thermal reprocessability.^18^ Since lignin contains many oxygen-containing functional groups, including phenolic hydroxyl groups and carboxylic acid groups, and it is weakly charged, it can chelate as a natural ligand with metal ions to form an ionomeric cluster.^19^

Inspired by this technique, several investigations on synthesizing and extruding high-performance renewable thermoplastics based on lignin/nitrile butadiene rubber (NBR) have already been reported by Naskar et al.^19–21^ In line with this, ZnCl_2_ was added to the lignin/NBR-based composites to construct the Zn^2+^–phenolic hydroxyl/carboxylate (from lignin) and Zn^2+^–cyano (from NBR) coordination interactions in the interphase of lignin and NBR, which imparts both improved compatibility and an enhanced reinforcing effect of lignin. In addition to that, poly­(ethylene oxide) (PEO) was also introduced as an interfacial adhesion promoter for the lignin in NBR.^22^ Although Akato et al. ended up with lignin/NBR-based composites with improved mechanical properties and good miscibility between the NBR and chemically unmodified lignin, in all these cases, lignin was mainly used as a secondary cofiller in combination with CB as an additional reinforcement or as a replacement for CB. Therefore, until now, the reinforcing potential of lignin as a sole biobased filler in an NBR matrix has not been fully explored, and very limited literature is available in which lignin alone has been incorporated into elastomers, particularly nitrile rubber, to evaluate its reinforcing efficiency. In this context, Agarwal et al. showed that 50 phr lignin filler level incorporated into a nitrile rubber exhibited 1.45 MPa tensile strength with 175% elongation at break (EB).^23^ Furthermore, Jiang et al. reported that incorporating less than 5 phr loading of colloidal lignin–cationic polyelectrolyte complexes into natural rubber latex followed by vulcanization resulted in no significant improvement in the mechanical properties when compared to the pristine vulcanized rubber.^24^ Moreover, many of these reports have used sulfur- or peroxide-based cross-linking agents to produce elastomers that are thermosets, which are not thermally reprocessable. Such traditional cross-linking methods are often accompanied by the emission of toxic volatile organic compounds during the cross-linking process. Hence, considerable efforts should be made to adopt a sustainable cross-linking strategy in manufacturing lignin-based elastomeric composites with high performance and good recyclability.^25,26^

Considering all of these aspects together, reactive compatibilization could be an effective alternative where both polymers possessing suitable functional groups can be chemically linked for better compatibility, fostering improved mechanical properties and stable morphologies. In this regard, oxazoline-functionalized polymers have proven their potential as reactive compatibilizers involving oxazoline rings in interfacial reactions due to the very high reactivity of the oxazoline group and, especially, its affinity toward nucleophiles.^27^ This has led to much finer and more stable morphologies, and this has had a dramatic effect in increasing interfacial adhesion even at lower concentrations of the oxazoline moiety. Because of this, the tensile strength and impact properties of these systems have been further improved.^27^

Inspired by this, in the present investigation, this oxazoline chemistry at the interface of lignin and NBR has been explored to investigate the reinforcing efficiency of lignin by analyzing its impact on the mechanical property of the as-prepared lignin/oxazoline-modified NBR composites. To achieve this, a facile and industrially viable melt-based route was adopted to oxazoline-modify a small percentage of the pendant nitrile (−C≡N) groups in the base NBR. This modification enables the −OH groups in lignin to chemically cross-link with the pendant oxazoline rings of the modified NBR, thereby enhancing the reinforcing capability. The oxazoline functionalization of NBR was performed in the melt in an internal mixer during processing prior to the addition of the additives, and the corresponding chemical conversion is shown in Figure 1a. Such a melt-based modification method is a more advantageous and sustainable approach to the traditional solution-based method, as it is relatively faster and involves no solvents. As per earlier reports, achieving a similar conversion percentage of the nitrile groups to oxazoline groups using the solution-based method requires a minimum of 5 h using a significant amount of solvents, such as chlorobenzene or o-dichlorobenzene, to dissolve the rubber, whereas the same degree of conversion can be achieved in about 30 min using the melt-based method.^27^

**Figure 1:**
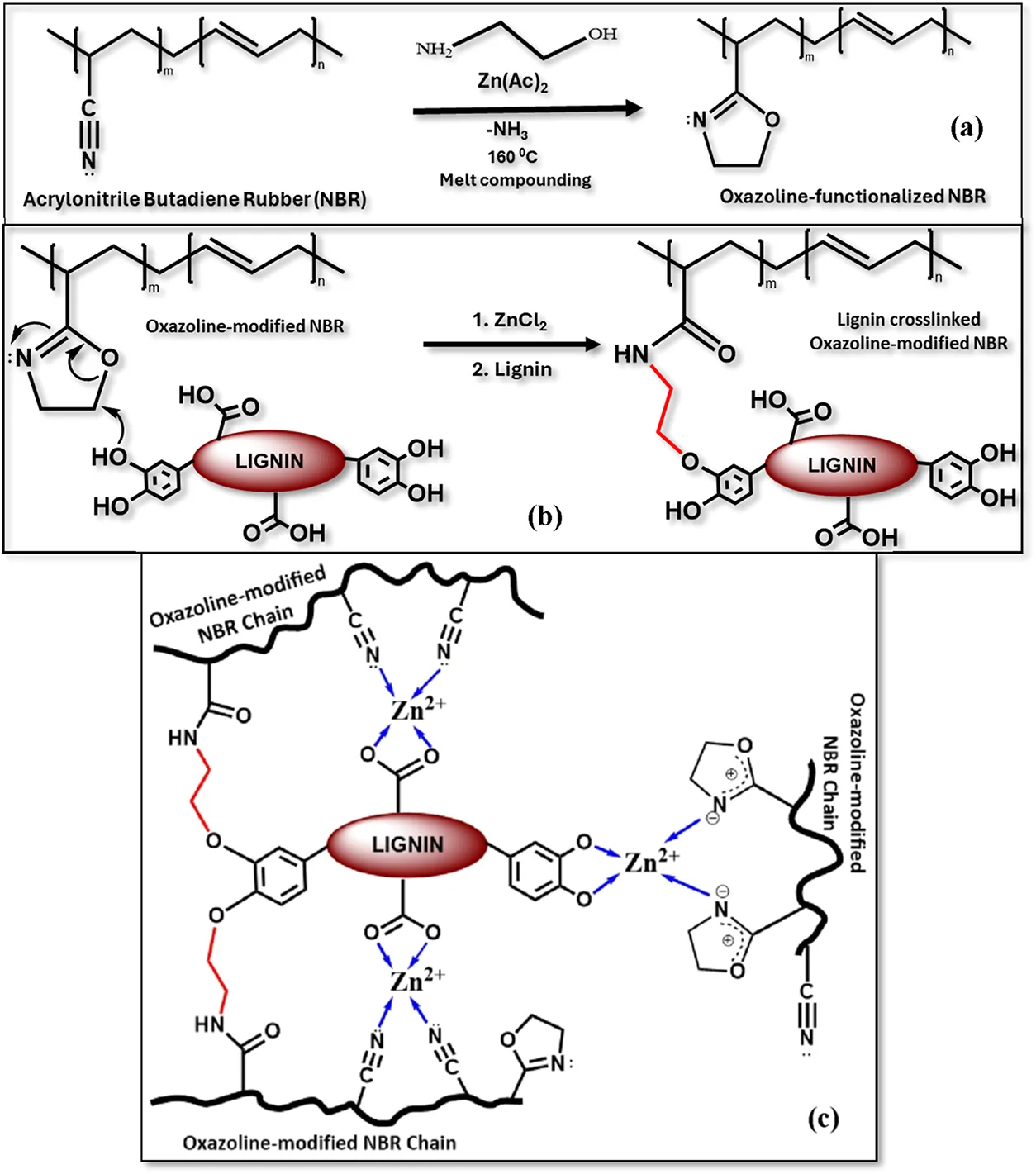
(a) Oxazoline modification of NBR, (b) cross-linking mechanism of lignin with oxazoline-modified NBR, and (c) formation of the double cross-linked network consisting of a permanent covalent bond (solid red colored bond) and sacrificial coordination bonds (blue-colored arrow) based on Zn^2+^–cyano/oxazoline and Zn^2+^–phenolic hydroxyl/carboxylate coordination.

Followed by the modification of the NBR, 4 phr ZnCl_2_ and 60 phr of lignin, mixed with polyethylene oxide (10 wt % of lignin content) were added for further processing. Therefore, the available −OH groups of lignin were anticipated to react with the pendant oxazoline group in the modified NBR to make permanent cross-linked networks, which is mechanistically shown in Figure 1b. The remaining −C≡N groups of NBR and −OH groups of lignin can be used to construct the Zn^2+^–cyano, Zn^2+^–phenolic hydroxyl, and Zn^2+^–carboxylate coordination interactions to form a double cross-linked network consisting of a permanent and a reversible dynamic network, as shown in Figure 1c. Therefore, the presence of permanent cross-links along with sacrificial bonds is expected to impart structural integrity together with recyclability into the final product.^28^ The selected volume fractions of lignin (60 phr) and ZnCl_2_ (4 phr) were determined by a detailed survey of the literature relating to NBR or XNBR/lignin/Zn^2+^ composites. Previous reports have indicated that increasing the loading of lignin content more than 60 phr does not result in any further significant improvement in tensile strength. A Zn^2+^ loading of 4 phr was found to be the optimum concentration to achieve the highest elastic recovery, while higher loadings led to a reduction in the percentage of elastic recovery.^16,19,29^ Lignin has already been investigated as a filler/cross-linker in a variety of resins, including phenolic,^30,31^ epoxy,^32–35^ polylactide,^36,37^ polyurethane,^38–40^ and PDMS resins.^3^

However, oxazoline ring-mediated cross-linking at the filler–polymer interface during the manufacturing of lignin/NBR-based composites without the use of conventional fillers, including CB or silica, and without traditional cross-linking agents, including sulfur and peroxide, has not yet been explored. Finally, the mechanical properties of the as-prepared oxazoline-modified NBR/lignin/ZnCl_2_-based composites were systematically compared with those of the unmodified NBR/lignin/ZnCl_2_-based composite to elucidate the effect of oxazoline functionalization of NBR on the resulting material properties.

## Materials

The high-performance elastomer acrylonitrile butadiene rubber (NBR) product-grade PERBUNAN-3945 F with 39 ± 1 wt % acrylonitrile content was supplied by ARLANXEO, USA. The cure characteristics of this NBR (specific gravity: 0.99) as reported in the technical data sheet of the as-received material based on IRB 7 is MDR 160 °C, microdies; ±0.5° arc, 30 min running time, and no preheat. Mooney Viscosity ML (1 + 4) 100 °C without pretreatment is 45 ± 5 MU. Purified lignin powder having >85% lignin content with a shelf life of 24 months was procured from Matexcel, USA. The catalyst zinc acetate [Zn­(Ac)_2_], antioxidant *N*-(1,3-dimethylbutyl)-*N*′-phenyl-p-phenylenediamine (6PPD 4020), 2-amino ethanol (AE), poly­(ethylene oxide) (PEO), and zinc chloride (ZnCl_2_) were procured from Sigma-Aldrich. All the chemicals were used in the as-received grade without further purification.

## Methods

### Oxazoline Modification of NBR

Varying weight percentages (5, 7.5, and 10% conversion of the −CN group to oxazoline) of the oxazoline-modified NBR were prepared by using a Brabender Meta-Station 16 internal mixer, according to the formulations listed in Table 1, and were designated as NOx-x. First, the NBR was charged into the mixer at 160 °C and masticated for 2 min at 60 rpm. Then, 2-amino ethanol (2.76 mol AE/mol of −CN) was dropwise added along with the catalyst (zinc acetate, 0.066 mol/mol −CN) and antioxidant (6PPD 4020, 0.1 wt % of polymer) as per the formulation table and reacted for a total of 30 min. At 10% conversion of the −CN group to oxazoline, excess amino ethanol was escaping from the mixer like drooling, so it was decided to not go beyond 10%. Torque data were monitored during mixing as an indicator of the extent of mixing and/or degree of the reaction. At the end of mixing, the samples were retrieved and stored at room temperature. All the mixtures were then molded in a Collins hydraulic press machine between two Teflon-coated flat-plaque molds at 160 °C and 15 MPa pressure for 6 min.

**Table 1:** Formulations (wt %) for Oxazoline Modification of NBR

code	NBR (g)	AE (mL)	Zn(Ac)_2_ (g)	6PPD (g)
NOx5	50	6.3	0.55	0.05
NOx7.5	50	10	0.65	0.05
NOx10	50	12	1.08	0.05

### Processing of Oxazoline-Modified NBR/Lignin Composites

The oxazoline-modified NBR/lignin composites were prepared using NOx-5 as the base rubber matrix using the Brabender Meta-Station 16 internal mixer. Compositions of the composites are listed in Table 2. First, the desired content of ZnCl_2_ was added to NOx5 at 60 rpm for 5 min. Then, a mixture of lignin and PEO (10 wt % of the lignin content) as the dispersion promoter for lignin was added and mixed for another 30 min at 160 °C. After mixing, the compounds were discharged from the mixer and molded using a Collins hydraulic press machine at 160 °C between two Teflon-coated flat-plaque molds for 20 min into 100 mm × 100 mm × 1 mm sheets at a pressure of 15 MPa.

**Table 2:** Formulations for Oxazoline-Modified NBR (NOx5)/ZnCl_2_/Lignin Composites

NOx5 (g)	lignin (g)	PEO (g)	ZnCl_2_ (g)	code
47.5			1.9	NOx5/Z4
32	19.2	1.92	1.3	NOx5/L60/Z4

### Characterization Techniques

The as-received lignin was characterized by Fourier transform infrared spectroscopy (FTIR), scanning electron microscopy (SEM), and thermogravimetric analysis (TGA). The mechanical properties of the as-prepared NOx5/lignin/ZnCl_2_ composites were evaluated and compared with those of unmodified NBR/lignin composites to assess their reinforcing potential beyond the use of lignin solely as a biobased filler. All of the experimental details and procedures of the adopted characterization techniques are explained below.

#### FTIR

FTIR spectra of lignin powder and the thin film of NBR and oxazoline-modified NBR of various percentages were recorded in the transmittance mode using a Bruker TENSOR 27. The spectra were collected with 64 sample scans and a resolution of 4 cm^–1^.

#### SEM

The morphology of the as-received lignin powder was investigated through scanning electron microscopy (SEM) in a Tescan Clara scanning electron microscope at 5 keV to check the physical appearance and determine the particle size of lignin. For SEM sample preparation, lignin powder was first dispersed in isopropyl alcohol by using an ultrasonic bath for 30 min. Then, a drop of the suspension was cast using a pipet onto a small piece of aluminum foil pasted on carbon tape on a stub and allowed it to dry. After drying, it was then sputtered with gold to make a conducting surface before being examined.

#### TGA

TGA of the as-received lignin powder was performed using a Mettler Toledo TGA instrument. Approximately 7 to 10 mg of lignin was placed in an alumina crucible, and the temperature was raised from 30 to 600 °C at a heating rate of 10 °C/min under a N_2_ atmosphere with a nitrogen flow of 50 mL/min.

#### Mechanical Testing

Tensile tests of the prepared composites were carried out using an AGS-X, Shimadzu tensile tester. Tests were carried out according to the ASTM. ASTM D638 Type V dumbbell test specimens were cut from 100 mm × 100 mm × 1 mm sheets, and at least five specimens were tested for each set. The tests were carried out at a crosshead speed of 500 mm/min using a 10 kN load cell with wedge grips.

## Results and Discussion

To check the availability of sufficient −OH groups at the surface of lignin, FTIR was carried out on the as-received lignin powder without any modification. The presence of the strong band around 3400 cm^–1^ in Figure 2a indicates the presence of −OH groups in lignin. The peak intensity ratios of the −C≡N group (2237 cm^–1^) from acrylonitrile, −C=N group in oxazoline (1664 cm^–1^), and −CH stretching at 2848 and 2920 cm^–1^ (*A*_2848_ and *A*_2920_) were measured by integrating the area under the corresponding peaks. The last two −CH stretching peaks were used as standard peaks, assuming their intensity to be unchanged as they are present in the nonreacting part of the molecule. In the oxazoline-modified NBR, the appearance of a strong peak at 1664 cm^–1^ corresponding to the C=N group in the oxazoline rings is observed in Figure 2b. Furthermore, the gradual decrease in the peak intensity of the nitrile group in NBR at 2237 cm^–1^ (*A*_2237_) and the simultaneous increase of the C=N peak intensity with an increase in AE and catalyst content from NOx5 to NOx10 are the signature of the oxazoline modification. The intensity ratio of these two peaks, specifically the *A*_1664_/*A*_2237_ ratio, was used as a crude estimation of the percentage conversion of oxazoline.^27^ Furthermore, the degree of oxazoline conversion was quantified as per eq 1 from the molar ratio of oxazoline rings and the sum of oxazoline and nitrile groups as below

1 $$D_{oxa}=\frac{\left[oxa\right]}{\left[oxa\right]+\left[C\equiv N\right]}\times 100\%=\frac{\left(\frac{A_{1664}}{A_{2920}+A_{2848}}\right)}{\left(\frac{A_{1664}}{A_{2920}+A_{2848}}\right)+\left(\frac{A_{2237}}{A_{2920}+A_{2848}}\right)}\times 100\%$$

**Figure 2:**
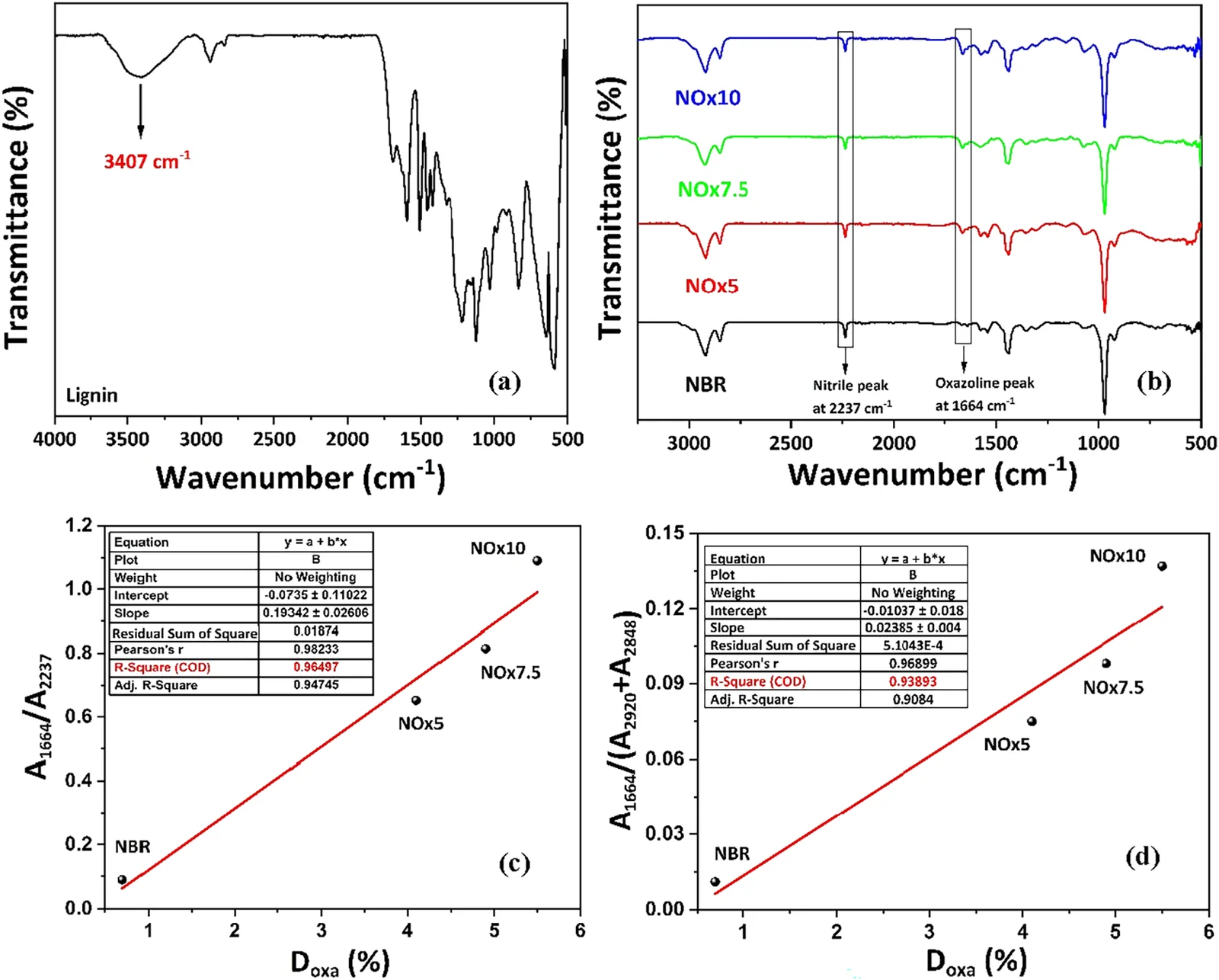
FTIR spectra of (a) as-received lignin and (b) different percentages of oxazoline-modified NBR, (c) correlation between the degree of modification and absorption ratio of C=N (*A*_1664_) to stretching C≡N (*A*_2237_), and (d) correlation between the degree of modification and absorption ratio of C=N (*A*_1664_) to stretching CH (*A*_2920_ + *A*_2848_).

The intensity ratio (*A*_1664_/*A*_2237_) is a linear function of the calculated degree of oxazoline conversion (*D*_oxa_) with a correlation coefficient of 0.96497 in Figure 2c. However, more reliable results are expected by correlating *D*_oxa_ with the intensity ratio of the C=N group (1664 cm^–1^) to the transmission of the bands coming from stretching of −C–H bonds (2920 + 2848 cm^–1^) in Figure 2d. In both cases, the correlation coefficients 0.96497 and 0.93893, respectively, are close to 1, indicating good linear correlations. However, deviation from linearity in higher loading of AE in NOx10 is clearly observed, which would imply that there is some limiting modification degree that can be achieved.^27^

The as-received base NBR samples containing 39 wt % acrylonitrile without any additives or shear history were prepared by compression molding at 160 °C and 15 MPa followed by cutting into 1 mm-thick dog bone-shaped samples (ASTM D638 Type V); they exhibited a tensile strength below 1 MPa. To verify the impact of the oxazoline modification on the base rubber’s mechanical properties, the mechanical properties of the unfilled oxazoline-modified rubbers were also measured as shown in Figure 3a, and the results are listed in Table 3. The unfilled rubbers show elongations exceeding 2500% without failure, and so the plotted *x*-axis was truncated at 1000% strain. Since these materials are clearly yielding at about 100% strain, after oxazoline modification, they are not yet cross-linked. The observed reduction in the tensile strength of NBR after oxazoline modification could be due to the chain scission or molecular weight degradation of NBR due to the shear degradation for prolonged mixing time. The NOx5 matrix among the other oxazoline-modified NBR was further selected as the base matrix for the preparation of the composites with lignin and other additives due to relatively higher tensile strength compared to NOx7.5 and consumption of total AE content without leaving unreacted content like NOx10.^41^

**Figure 3:**
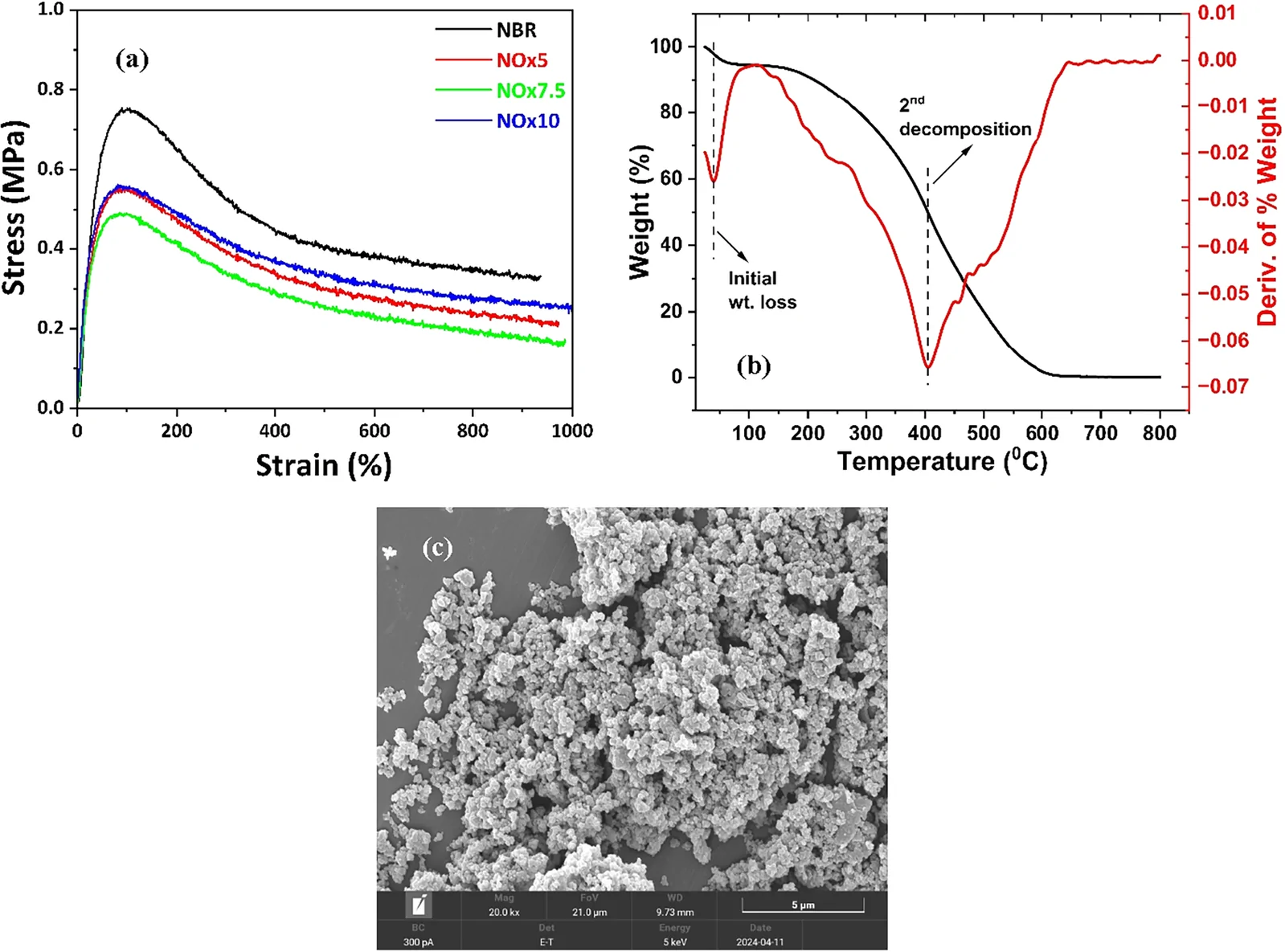
(a) Tensile stress vs strain plot of NBR and various percentages of oxazoline-modified NBR, (b) combined plot of wt % and derivative wt % vs temperature of lignin, and (c) FESEM image of the as-received lignin powder.

**Table 3:** Mechanical Properties of Base NBR and Oxazoline-Modified NBR

samples	tensile strength (MPa)
NBR	0.75 ± 0.05
NOx5	0.56 ± 0.01
NOx7.5	0.49 ± 0.03
NOx10	0.55 ± 0.01

Since the modification and subsequent mixing (with additives) of the oxazoline-modified NBR were carried out in an internal mixer at 160 °C, it was essential to evaluate the thermal stability of the lignin to determine whether it could withstand this processing temperature without undergoing significant degradation. Therefore, thermogravimetric analysis at a temperature range of 35–800 °C was carried out on the as-received lignin powder as shown in Figure 3b combined with the derivative plot of the remaining wt % vs temperature as a double *y* axis. The initial weight loss observed in the range of 40–135 °C as shown in Figure 3b is primarily attributed to the removal of any moisture and volatile components present in the sample during the initial heating stage.^42–44^ The second decomposition of the β-O-4 linkage observed around 400 °C is associated with the breakdown of the carbohydrates from lignin samples, which are transformed into volatile gases such as CO, CO_2_, and CH_4_.^45^

The surface morphology of the as-received lignin powder was also investigated using FESEM analysis. It shows that the particle size ranges from 2 to 5 μm, with each particle composed of finer particles and exhibiting a more defined structured as depicted in Figure 3c. The observed agglomerated state in the micron range (2–5 μm) of the lignin particles could be ascribed to the intermolecular hydrogen bonding between macromolecular domains.^46^ Based on these characteristics, the material can be expected to possess similar reinforcing properties.^47^

The mechanical properties of the as-prepared oxazoline-modified NBR (NOx5)/ZnCl_2_/lignin-based composites were investigated and compared to those of the unmodified NBR/ZnCl_2_/lignin-based composites. The corresponding tensile stress vs strain curves of NBR and oxazoline-modified NBR-based composites are presented in Figure 4a,b, respectively, and the results are listed in Table 4.

**Figure 4:**
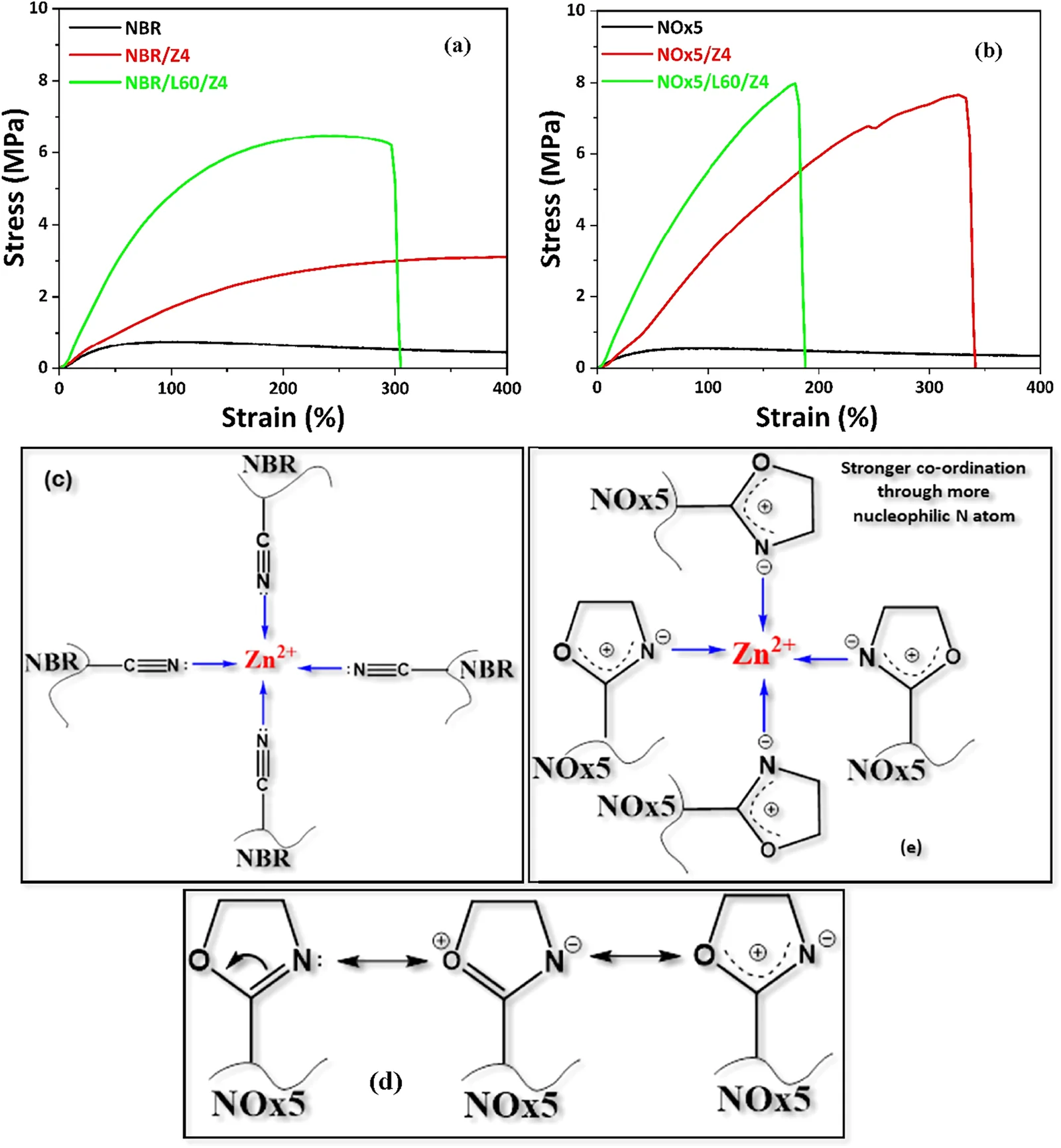
Tensile stress vs strain plots of (a) NBR/lignin/ZnCl_2_ composites, (b) NOx5/lignin/ZnCl_2_ composites, (c) Zn^2+^–cyano coordination interactions (blue-colored arrow) for the NBR/Z4 composite, (d) resonance structures of delocalized iminic double bond electron density around the N–C–O center of the oxazoline ring in NOx5, and (e) Zn^2+^–oxazoline coordination interactions (blue-colored arrow) for the NOx5/Z4 composite.

**Table 4:** Mechanical Properties of NBR/Lignin/ZnCl_2_ and NOx5/Lignin/ZnCl_2_ Composites

samples	codes	tensile strength (MPa)	elongation at break (%)
NBR composites	NBR	0.75 ± 0.05	
	NBR/Z4	3.48 ± 0.45	900 ± 180
	NBR/L60/Z4	6.48 ± 0.55	304 ± 38
oxazoline-modified NBR composites	NOx5	0.55 ± 0.01	
	NOx5/Z4	7.63 ± 0.50	340 ± 37
	NOx5/L60/Z4	7.97 ± 0.80	186 ± 14

The control base elastomer samples of both raw NBR and NOx5 without a filler and cross-linker or curatives exhibited very low tensile strength below 1 MPa and an elongation of up to 2500 and 1000% for NBR and NOx5, respectively (*x*-axis has been truncated at 400%). Incorporating a small amount (4 phr) of ZnCl_2_ in either NBR or NOx5 during mixing, followed by molding, led to an increase in the tensile strength and elastic modulus and a reduction in elongation at break % for both cases. For the unmodified NBR sample NBR/Z4, Zn^2+^ can link nitrile groups of NBR through metal ligand coordination sacrificial bonds (as shown in Figure 4c), thereby leading to an enhanced tensile strength (3.48 ± 0.46 MPa) and a reduced elongation at break % (900 ± 180%). However, for the oxazoline-modified NBR sample (NOx5/Z4), the oxazoline ring can coordinate more strongly with the Zn^2+^ due to the delocalized iminic double bond electron density around the N–C–O centers, as illustrated by the resonance structures in Figure 4d.^48^ Accordingly, stronger metal–ligand coordination bonds are formed via the more nucleophilic N atom in the oxazoline ring in comparison to the N atom in the pendant nitrile group of unmodified NBR as shown in Figure 4e. Incorporating 4 phr ZnCl_2_ into the NOx5 formulation resulted in more rigid specimens exhibiting enhanced tensile strength (7.63 ± 0.5 MPa) and modulus, accompanied by a reduction in elongation at break (340 ± 36%). Notably, the NOx5/Z4 composite demonstrated an approximately 120% increase in tensile strength. This improvement exceeds that observed for the unmodified NBR composites (NBR/L60/Z4) containing 4 phr of ZnCl_2_ and 60 phr of lignin. These findings demonstrate that physical cross-links formed through the rational selection of metal and ligand coordination sacrificial bonds can lead to significant reinforcement in the elastomer matrix even without a filler. Wang et al.^16^ reported a formulation using NBR N220S (acrylonitrile content: 41 wt %) with 1.5 phr of sulfur, which exhibited a tensile strength of 4.1 ± 0.2 MPa and an elongation at break of 367 ± 15%. For another formulation, 10 phr of ZnCl_2_ was added along with 1.5 phr of sulfur, showing 5.2 ± 0.2 MPa tensile strength and 282 ± 10% elongation at break.^16^ Compared to with these reports, the current NOx5/Z4 composite with only 4 phr of ZnCl_2_ loading shows a significantly higher tensile strength (7.63 ± 1.5 MPa), further justifying the positive impact of the oxazoline modification of NBR on mechanical properties.

To achieve a high reinforcing efficiency of fillers in elastomer matrices and enhanced mechanical performance, three key factors must be considered: (1) a filler size preferably below approximately 1 μm, (2) uniform dispersion of the fillers within the elastomer matrix, and (3) strong interfacial interactions between the filler and the polymer matrix.^29,49–51^ A high-shear melt-phase mixing strategy (at 60 rpm) was adopted to further reduce the size of lignin domains and improve their dispersion in the polymer matrices.^29,52,53^

In an attempt to utilize ultrahigh lignin loadings in elastomer matrices, 60 phr of lignin was added into both NBR and NOx5 as per the formulations shown in Table 1. For the unmodified NBR sample (NBR/L60/Z4), the addition of 60 phr of lignin led to an improvement of 86% in tensile strength (6.48 ± 0.55 MPa) compared to NBR/Z4 and a reduction in elongation at break (303 ± 38%). The NOx5/L60/Z4 composite increased its tensile strength (7.97 ± 0.85 MPa), which was even higher, ∼129% with respect to NBR/Z4, as shown in Figure 5a. This enhancement can primarily be attributed to the dual function of lignin both as a reinforcing filler and a cross-linking agent within the NOx5 elastomer. First, this effect arises from the formation of a double cross-linked network comprising permanent covalent bonds and reversible sacrificial bonds, as previously illustrated in Figure 1c. Second, strong filler–matrix interfacial interactions were developed via the oxazoline ring-opening reaction of the NOx5 by the nucleophilic attack of the −OH group of lignin (mechanistic pathway is shown in Figure 1b), which could potentially also improve the dispersion of lignin in the elastomer matrix. Third, the Zn^2+^–ligand coordination bonds function as sacrificial interactions that preferentially dissociate under applied stress, thereby dissipating mechanical energy during deformation. This energy dissipation mechanism contributes to the enhanced toughness and improved tensile strength of the composite. Moreover, the significant improvements in the modulus of the samples along with a reduction of the elongation at break could be attributed to the synergistic contribution of the coordination effect of lignin endowed with its dual role as a rigid phase reinforcing filler hindering the rubber chain movement and the reactive cross-linking component with oxazoline-modified NBR.^2,29^ Comparison of the presented results with previously reported data by Wang et al.^16^ using NBR N220S (acrylonitrile content: 41 wt %) with 1.5 phr of sulfur and 40 phr of lignin reported a tensile strength of 3.9 MPa at 300% elongation at break. This value is significantly lower than that obtained from the prepared NBR/L60/Z4 composite (6.48 ± 0.55 MPa), even in the absence of oxazoline modification. In another work, Naskar et al.^41^ examined NBR-33 (33 wt % acrylonitrile content) containing 120 phr of hardwood soda lignin and softwood kraft lignin in the presence of 2.5 phr of dicumyl peroxide. The reported tensile strengths were 4.63 ± 0.80 and 8.82 ± 1.63 MPa, corresponding to elongations at break of 227 ± 80 and 126 ± 35%, respectively. In comparison to these results, the current NBR/L60/Z4 composite exhibited relatively higher tensile strength (6.48 ± 0.55 MPa) at a lignin loading of 60 phr in the presence of 4 phr of ZnCl_2_. Furthermore, the oxazoline-modified NBR-based composites NOx5/Z4 (7.63 ± 0.50 MPa) and NOx5/L60/Z4 (7.97 ± 0.80 MPa) demonstrated comparable tensile performances. These results were achieved through a sustainable cross-linking strategy involving covalent cross-linking between lignin and oxazoline-modified NBR at relatively low lignin loadings (60 phr), combined with the formation of reversible Zn^2+^–ligand sacrificial coordination bonds. Notably, this strategy eliminates the need for peroxide- or sulfur-based cross-linkers as illustrated in Figure 5b.

**Figure 5:**
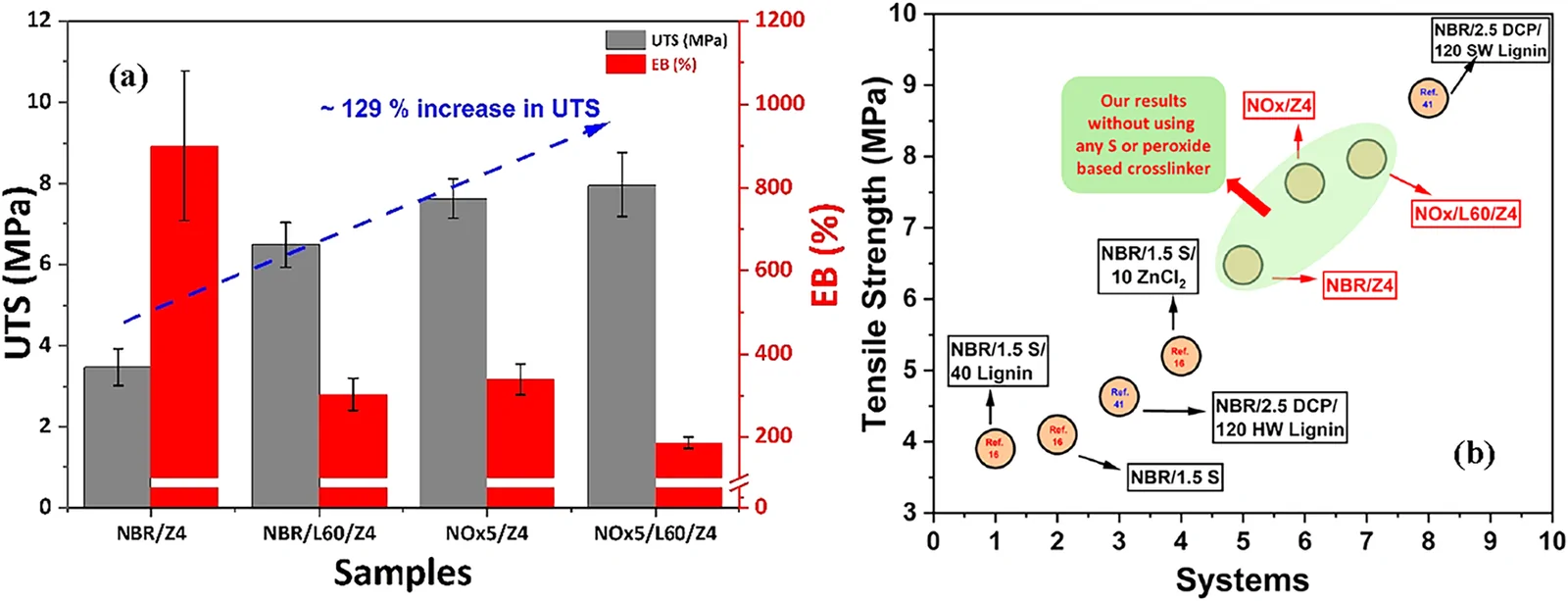
(a) Ultimate tensile strength (UTS) and percentage elongation at break (EB) of NBR/Z4, NBR/L60/Z4, NOx5/Z4, and NOx5/L60/Z4 composites and (b) benchmark study comparing the tensile strength of current composites with earlier reported literature data (reference numbers are as per in the manuscript) on NBR-based similar systems.

The key formulation differences of the systems in the benchmark study and plot (Figure 5b), including NBR grade, lignin loading, cross-linking system, and testing standard, are tabulated in Table 5 for comparison. These comparative results further demonstrate that oxazoline modification of NBR significantly influences its mechanical performance, yielding a tougher elastomer characterized by increased tensile strength and modulus, albeit with reduced extensibility, in the absence of conventional sulfur- or peroxide-based permanent cross-linkers. Moreover, these findings support our earlier assertion that the presence of ZnCl_2_ promotes improved lignin dispersion with the NBR matrix. Overall, the mechanical properties of such NBR/lignin/ZnCl_2_-based composites can be tailored over a broad performance window by systematically varying the degree of oxazoline modification and filler loading, thereby enabling material optimization for specific requirements.

**Table 5:** Key Formulation Differences of the Systems Considered in the Benchmark Study

system	NBR grade	lignin loading	cross-linker concentration	cross-linking system	testing standard	references
NBR/1.5S/40 lignin	NBR-220S (acrylonitrile content 41 wt %)	40 phr	sulfur, 1.5 phr	sulfur-based cross-linking	GB/T528–2009 at an elongation speed of 200 mm/min at 25 °C	^16^
NBR/1.5S	Do		sulfur, 1.5 phr	Do	Do	^16^
NBR/2.5DCP/120 HW lignin	NBR (Krynac 3330F, 33% acrylonitrile content)	hardwood soda lignin, 120 phr	DCP, 2.5 phr	peroxide-based cross-linking	ASTM-D412 at a crosshead speed of 500 mm/min	^41^
NBR/1.5S/10 ZnCl_2_	NBR-220S (acrylonitrile content 41 wt %)		sulfur, 1.5 phr	sulfur-based cross-linking	Do	^16^
NBR/Z4	NBR PERBUNAN-3945 F with 39 ± 1 wt % acrylonitrile content			Zn^2+^ coordination-based cross-linking	ASTM-D638 type V at a crosshead speed of 500 mm/min	present work
NOx5/Z4	Do			Zn^2+^ coordination-based cross-linking	Do	present work
NOx5/L60/Z4	Do	60 phr		dual cross-linking based on Zn^2+^ coordination and oxazoline-mediated reactive mixing with lignin	Do	present work
NBR/2.5DCP/120 SW lignin	NBR (Krynac 3330F, 33% acrylonitrile content)	softwood kraft lignin, 120 phr	DCP, 2.5 phr	peroxide-based cross-linking	ASTM-D412 at a crosshead speed of 500 mm/min	^41^

## Conclusions

In the present work, a sustainable cross-linking strategy was developed to integrate lignin into an oxazoline-modified nitrile butadiene rubber (NBR) matrix, enabling lignin to function simultaneously as a reinforcing filler and a cross-linking agent. A small fraction of the pendant nitrile groups of NBR was converted into oxazoline rings via an industrially viable, solvent-free melt-processing approach. This modification ensured sustainability while maintaining process scalability. Furthermore, the incorporation of ZnCl_2_ facilitated the formation of metal–ligand coordination interactions, including Zn^2+^–phenolic hydroxyl/carboxylate (from lignin) and Zn^2+^–cyano/oxazoline (from NBR/NOx) complexes. These dynamic interactions enhanced interfacial adhesion and improved the dispersion of lignin within the NBR/NOx matrix, thereby maximizing its reinforcing efficiency. The resulting elastomeric composites exhibited a dual cross-linked network comprising permanent covalent bonds (via oxazoline–lignin reactions) and reversible sacrificial metal–coordination bonds. This synergistic network architecture imparted improved structural integrity and enhanced mechanical performance. Notably, the NOx5/L60/Z4 composite demonstrated a tensile strength of 7.97 ± 0.85 MPa and an elongation at break of 185 ± 14%, representing an approximately 23% increase in tensile strength compared to the unmodified NBR/ZnCl_2_/lignin-based composites (6.48 ± 0.55 MPa) at the same lignin (60 phr) and ZnCl_2_ (4 phr) loadings. The inferior performance of the unmodified system is attributed to the presence of only reversible Zn^2+^-mediated coordination bonds between NBR and lignin, in the absence of covalent interfacial cross-links. Overall, oxazoline modification significantly enhances the reinforcing capability of lignin by promoting covalent interfacial cross-linking in addition to dynamic metal–ligand interactions. This study provides a promising strategy for exploiting oxazoline chemistry to improve lignin–elastomer compatibility and interfacial interactions, enabling high-volume incorporation of lignin as a renewable alternative to petroleum-derived fillers such as carbon black. Consequently, this approach offers a sustainable pathway for the efficient development of high-performance lignin-based elastomeric composites.
